# Peripheral artery disease in peritoneal dialysis and hemodialysis patients: single-center retrospective study in Taiwan

**DOI:** 10.1186/1471-2369-13-100

**Published:** 2012-09-03

**Authors:** Chun-Chuan Lee, Chih-Jen Wu, Li-Hua Chou, Su-Mei Shen, Sheng-Fang Chiang, Pi-Chu Jen, Mei-Ching Yeh, Chi-Feng Pan

**Affiliations:** 1 Division of Endocrinology and Metabolism, Department of Internal Medicine, Mackay Memorial Hospital, Taipei, Taiwan; 2 Division of Nephrology, Department of Internal Medicine, Mackay Memorial Hospital, Taipei, Taiwan; 3 Division of Hemodialysis and Peritoneal Dialysis, Department of Nursing, Mackay Memorial Hospital, Taipei, Taiwan; 4 College of Medicine, Taipei Medical University, Taipei, Taiwan; 5 Mackay Medicine, Nursing and Management College, Taipei, Taiwan

**Keywords:** Ankle-brachial pressure index (ABI), Cardiovascular disease, End-stage renal disease, Hemodialysis, Peritoneal dialysis, Peripheral artery disease

## Abstract

**Background:**

Peripheral artery disease (PAD) is a condition characterized by restricted blood flow to the extremities, and is especially common in the elderly. PAD increases the risk for mortality and morbidity in patients with end-stage renal disease (ESRD), especially those on hemodialysis (HD).

**Methods:**

The records of 484 patients with end-stage renal disease who were on HD or peritoneal dialysis (PD) were reviewed. PAD was diagnosed based on the ankle-brachial pressure index (ABI). Demographic and clinical characteristics were analyzed.

**Results:**

PAD had an overall prevalence of 18.2% and was significantly more common in HD patients (21.8%) than in PD patients (4.8%). Advanced age, diabetes mellitus, smoking, low parathyroid hormone level, elevated serum ferritin, elevated serum glucose, and low serum creatinine levels increased the risk for PAD. PAD was independently associated with advanced age, diabetes mellitus, duration of dialysis, low serum creatinine, and hyperlipidemia. PD patients had a significantly lower prevalence of PAD than HD patients, maybe due to their younger age and lower prevalence of diabetes mellitus in this present study.

**Conclusions:**

The prevalence of PAD was greater in the HD group than the PD group. Most of the risk factors for PAD were specific to HD, and no analyzed factor was significantly associated with PAD in PD patients.

## Background

Cardiovascular disease is the most common cause of death in patients with end-stage renal disease (ESRD) [1]. Peripheral artery disease (PAD) is an important marker of systemic atherosclerosis, and often leads to significant morbidity and mortality, particularly in patients with ESRD [2]. The National Health and Nutrition Examination Survey (NHANES) of 1999–2000 reported that PAD affects approximately 5 million adults, including 12-20% of Americans aged 65 years and older [3]. The prevalence of PAD increases dramatically with age, but the actual prevalence may be underestimated. Experts have estimated that PAD affects approximately 8 million people in the U.S. alone [4]. In contrast, little is known about the prevalence of PAD in Asian ESRD patients.

Despite the high prevalence of PAD and its association with increased cardiovascular disease, few PAD patients are given treatment for this condition, because most are asymptomatic [5]. PAD often progresses silently until the onset of the most common symptom, intermittent claudication [2]. However, in the general population, only about 10% of patients with PAD develop this classic symptom, about 40% have no complaint of leg pain, and about 50% have diverse leg symptoms that are not typical of classic claudication [4].

PAD is considered a marker for systemic atherosclerotic disease. Patients with PAD have a four-to-five fold greater risk of dying from a cardiovascular event and a two-to-three fold higher overall mortality rate relative to patients without PAD [6]. The irreversible and reversible risk factors associated with PAD are similar to those for ischemic heart disease [7]. Although younger patients represent a small percentage of PAD cases, they have poorer long-term outcome and experience more complications associated with bypass revascularization surgery [6]. There is a remarkably high prevalence of PAD in patients with renal insufficiency [8]. In particular, 24% of the NHANES population older than 40 years with renal insufficiency had PAD, but only 3.7% with normal renal function had PAD, and this association was independent of diabetes, hypertension, and age [8].

Increased duration of dialysis and conventional cardiovascular disease risk factors (other than hypertension and hyperlipidemia) are known risk factors for PAD [8]. PAD has been diagnosed in 25.3% of ESRD patients, and the prevalence varied widely among different populations [5]. A recent study of patients on maintenance ambulatory peritoneal dialysis (PD) indicated the prevalence of PAD was 27.4% of all patients, and was 45% in patients older than 70 years [9].

The ankle-brachial pressure index (ABI) is the simplest, least expensive, and most reliable method of detecting atherosclerosis in asymptomatic individuals with suspected PAD [10]. Numerous clinical and epidemiological studies have used the ABI to screen for PAD because PAD of the lower extremities is associated with poor overall prognosis [11,12]. In the U.S., the prevalence of PAD is much higher in patients on hemodialysis (HD) than in age- and sex-matched healthy subjects [11]. A Finnish study indicated the prevalence of an elevated ABI was 8.4% among patients referred for vascular consultation and that PAD was much more common in patients with renal failure [13]. However, there is little available data on relationship of PAD and renal failure in Asian populations, and fewer data in PD patients.

In the present study, we compared HD and PD patients who were receiving maintenance dialysis in the Mackay Memorial Hospital (Taipei, Taiwan) to identify the prevalence and risk factors associated with PAD in ESRD patients and to compare prevalence and risk of PAD in HD and PD patients.

## Methods

Between June 1, 2007 and September 30, 2007, 529 patients with ESRD were on maintenance dialysis in the Dialysis Unit of Mackay Memorial Hospital. A total of 45 patients had severe illnesses, such as liver cirrhosis, malignancies, apparent acute inflammatory diseases, or amputation of both legs and were excluded. ABI was recorded for the remaining 484 patients, which included 380 HD patients (all with synthetic membranes) and 104 PD patients (median [IQR] urea clearance, 2.27 L/Day [0–20.52]). The Institutional Review Board of Mackay Memorial Hospital approved this retrospective study of the records of these patients. All patients had undergone dialysis for at least 3 months prior to enrollment.

The following basic data was recorded for all subjects at baseline: age, gender, height, weight, BMI (kg/m^2^), and duration and mode of dialysis. Analysis of risk factors associated with PAD considered the effect of smoking (ever versus never), cardiovascular or cerebrovascular disease (CVD), hypertension, diabetes, hyperlipidemia, and underlying renal disease. Dialysis dose was calculated from Daugirdas formula for Kt/V and serum assays were performed for intact parathyroid hormone (iPTH), hematocrit (Hct), fasting glucose, blood urea nitrogen (BUN), creatinine (Cr), calcium (Ca), phosphorus (P), calcium and phosphorus product (Ca×P), albumin, cholesterol, triglyceride (TG), total carbon dioxide (TCO_2_), and ferritin. Both groups were given the minimal recommended doses of dialysis and the presented Kt/V results indicate the Kt/V per dialysis session.

The ABI ratio was calculated with the Fukuda Vascular Screening system (VaSera VS-1000™, Fukuda Denshi Co., Ltd., Tokyo, Japan) and were performed while the subjects were supine. Systolic blood pressure was measured at the brachial artery on the nondialytic vascular access arm and on both ankles (at the posterior tibial arteries). PAD was diagnosed if ABI was less than 0.9 in either lower extremity. A patient was assigned a score of “PAD-one” if the ABI was low in one lower extremity and a score of “PAD-two” if the ABI was low in both lower extremities.

### Statistical analysis

For continuous variables, data are presented as means ± standard deviations (SDs) and an independent two-sample *t*-test was used to compare the HD and PD groups. If variables were not normally distributed, data is presented as medians and interquartile ranges (IQRs) and the Mann–Whitney U test was used for comparisons. For categorical variables, data are presented as number (percentage) and the Chi-square test was used for comparisons of the two binary variables. Logistic regression analysis was used to identify factors related to the presence of PAD. Variables associated with PAD based on univariate analysis were entered into multivariate models. All statistical assessments were two-sided and a *p*-value less than 0.05 was considered significant. Statistical analyses were performed using SPSS 15.0 (SPSS Inc., Chicago, IL).

## Results

Table 1 shows the demographic and clinical characteristics of the 484 ESRD patients who were on HD or PD in the dialysis unit of Mackay Memorial Hospital from June 1, 2007 to September 30, 2007. There were 213 males and 271 females, the mean age was 57.91 ± 13.64 years (range: 21 to 90 years), and there were 380 patients undergoing HD (78.5%) and 104 patients undergoing PD (21.5%). The overall prevalence of PAD was 18.2% (n = 88). There were statistically significant differences between the two groups with regard to age (HD: 60.39 ± 12.81 years, PD: 48.87 ± 12.76 years, *p*<0.001), median duration of dialysis (HD: 55 months, PD: 36 months, *p*<0.001), history of DM (HD: 36.6%, PD: 18.3%, *p*<0.0001), smoking status (HD: 36.6%, PD: 14.4%, *p* = 0.003), and in the overall prevalence of PAD (HD: 21.8%, PD: 4.8%). The prevalence of PAD in one extremity (HD: 11.8%, PD: 1.9%) and in two extremities (HD: 10.0%, PD: 2.9%) were greater in the HD group ( *p*<0.001) In addition, the HD group had significantly higher levels of Hct, albumin, ferritin, uric acid ( *p*<0.05 for all) and significantly lower levels of Kt/V per dialysis session, TCO_2_, cholesterol, blood sugar, Cr, Ca, P and Ca×P (*p*<0.05 for all).

**Table 1:** Clinical and demographic characteristics of hemodialysis (HD) and peritoneal dialysis (PD) patients (n = 484)

**Variables**	**Total (n=484)**	**HD group (n=380)**	**PD group (n=104)**	**P-value**
Age (years)^1^	57.91±13.64	60.39±12.81	48.87±12.76	<0.001*
Gender, n (%)^2^				0.131
Male	213 (44.0)	174 (45.8)	39 (37.5)	
Female	271 (56.0)	206 (54.2)	65 (62.5)	
BMI (kg/m^2^)^1^	22.49±3.43	22.44±3.34	22.67±3.77	0.533
Duration of Dialysis (months)^3^	48 (26, 88)	55 (31, 94)	36 (14, 57)	<0.001*
DM, n (%)^2^	158 (32.6)	139 (36.6)	19 (18.3)	<0.001*
HT, n (%)^2^	356 (73.6)	280 (73.7)	76 (73.1)	0.901
Hyperlipidemia, n (%)^2^	199 (41.1)	149 (39.2)	50 (48.1)	0.103
Smoking, n (%)^2^	124 (25.6)	109 (28.7)	15 (14.4)	0.003*
PAD, n (%)^2^				<0.001*
Non	396 (81.8)	297 (78.2)	99 (95.2)	
PAD-one	47 (9.7)	45 (11.8)	2 (1.9)	
PAD-two	41 (8.5)	38 (10.0)	3 (2.9)	
Daugirdes Kt/V per dialysis session^1^	1.90±0.40	1.81±0.35	2.22±0.41	<0.001*
Hct (%)^1^	30.10±4.36	30.37±4.12	29.13±5.04	0.023*
TCO_2_ (mmol/L)^1^	23.54±2.87	22.71±2.32	26.60±2.60	<0.001*
Albumin (g /dL)^1^	3.94±0.38	3.97±0.37	3.80±0.39	<0.001*
i-PTH (pg/ mL)^3^	284.8 (163.4, 480.7)	281.7 (166.2, 468.9)	328.9 (160.7, 512.0)	0.541
Ferritin (ng/ mL)^3^	573.0 (364.0, 761.7)	632.8 (487.5, 799.6)	330.7 (173.1, 482.1)	<0.001*
Cholesterol (mg/dL)^3^	183 (154, 209)	179 (151, 205)	190 (172, 213)	0.012*
TG (mg/ dL)^3^	125 (82, 195)	124 (82, 194)	130 (82, 232)	0.372
Fasting blood sugar (mg/dL)^3^	95 (83, 117)	90 (81, 117)	104 (95, 116)	<0.001*
Cr (mg/dL)^1,4^	11.01±2.77	10.61±2.44	12.44±3.40	<0.001*
Uric Acid (mg/dL)^1^	7.23±1.30	7.35±1.31	6.80±1.15	<0.001*
Ca (mg/dL)^1^	9.07±0.83	8.97±0.79	9.44±0.87	<0.001*
P (mg/dL)^1^	5.41±1.38	5.32±1.37	5.73±1.36	0.006*
Ca×P (mg/dL)^1^	49.16±13.64	47.78±13.26	54.15±13.90	<0.001*

* significant difference between groups (p<0.05).

P-values were calculated by: ^1^Independent two sample *t*-test (mean ± SD); ^2^Chi-square test (number [percentage]); ^3^Mann-Whitney U test (median [IQR]); ^4^Cr was measured pre-dialysis.

**Figure 1:**
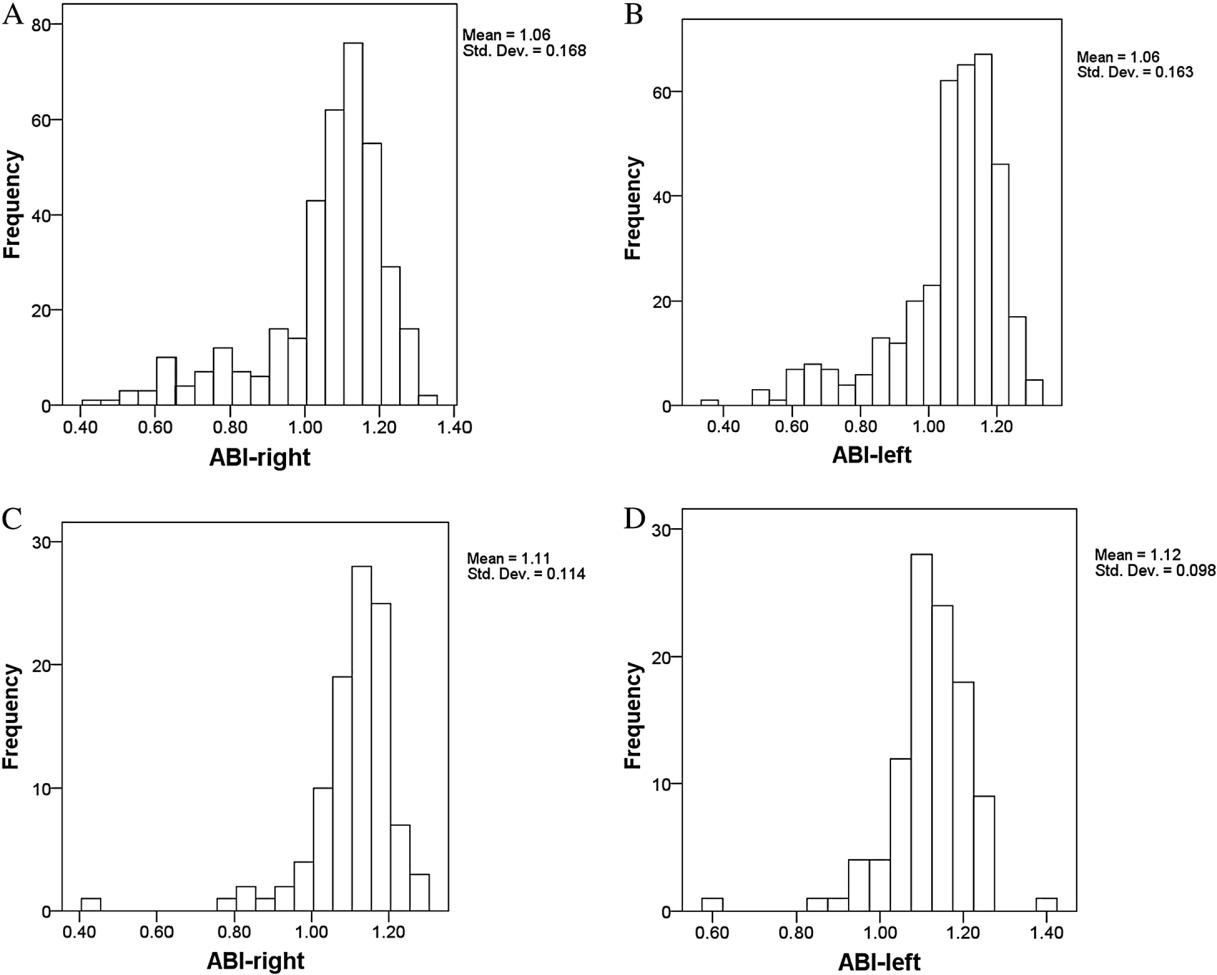
Distribution of ABI measurements on the right and left side of hemodialysis patients (A and B) and peritoneal dialysis patients (C and D).

Figure 1 shows the frequency distributions of our ABI measurements. There were no significant differences in the mean ± SD of the left and right ABI among HD patients or in the mean ± SD of the left and right ABI among PD patients. However, HD patients had a significantly lower mean ABI (*p* = 0.047).

Table 2 shows the demographic and clinical characteristics of ESRD patients with and without PAD. PAD patients were more likely to have diabetes mellitus (61.4% *vs* 26.3%, *p*<0.001), older (68.66 ± 10.54 years *vs* 55.52 ± 13.10 years, *p* <0.001), smoke (35.2% *vs* 23.5%, *p* = 0.022). Patients without PAD had higher levels of serum albumin, i-PTH, and Cr ( *p*<0.05 for all) and patients with PAD had higher levels of ferritin and blood sugar ( *p*<0.05 for both).

**Table 2:** Clinical and demographic characteristics of all maintenance dialysis patients with and without peripheral arterial disease (PAD) (n = 484)

**Variables**	**Non-PAD (n=396)**	**PAD (n=88)**	**P-value**
Age (years)^1^	55.52±13.10	68.66±10.54	<0.001*
Gender, n (%)^2^			0.136
Male	168 (42.4)	45 (51.1)	
Female	228 (57.6)	43 (48.9)	
BMI (kg/m^2^)^1^	22.53±3.56	22.30±2.82	0.566
Type of dialysis, n (%)^2^			<0.001*
HD	297 (75.0)	83 (94.3)	
PD	99 (25.0)	5 (5.7)	
Duration of Dialysis(months)^3^	47 (25, 86)	57 (27, 96)	0.318
DM, n (%)^2^	104 (26.3)	54 (61.4)	<0.001*
HT, n (%)^2^	292 (73.7)	64 (72.7)	0.846
Hyperlipidemia, n (%)^2^	158 (39.9)	41 (46.6)	0.248
Smoking, n (%)^2^	93 (23.5)	31 (35.2)	0.022*
Daugirdes Kt/V^1^	1.91±0.39	1.85±0.46	0.218
Hct (%)^1^	30.02±4.50	30.44±3.66	0.418
TCO_2_ (mmol/L)^1^	23.55±2.95	23.51±2.45	0.897
Albumin (g /dL)^1^	3.96±0.38	3.84±0.38	0.012*
i-PTH (pg/ mL)^3^	309.1(173.1, 497.8)	223.6 (101.8, 385.6)	0.002*
Ferritin (ng/ mL)^3^	559.2 (354.0, 754.3)	651.3 (479.1, 846.8)	0.017*
Cholesterol (mg/dL)^3^	183 (154.5, 204.5)	178 (150, 216)	0.915
TG (mg/ dL)^3^	122 (82, 192)	137 (81, 213)	0.398
Blood sugar (mg/dL)^3^	93 (82, 112)	107 (85, 159)	<0.001*
Cr (mg/dL)^1^	11.38±2.67	9.33±2.61	<0.001*
Uric Acid (mg/dL)^1^	7.27±1.29	7.05±1.33	0.142
Ca (mg/dL)^1^	9.07±0.84	9.07±0.78	0.990
P (mg/dL)^1^	5.46±1.35	5.16±1.51	0.064
Ca×P (mg/dL)^1^	49.66±13.42	46.84±14.47	0.081

* significant difference between groups (p < 0.05).

P-values were calculated by: ^1^Independent two sample *t*-test (mean ± SD); ^2^Chi-square test (number [percentage]); and ^3^Mann-Whitney U test (median [IQR]).

Table 3 shows the results of univariate and multivariate logistic regression analysis of factors associated with PAD in all of the maintenance dialysis patients. Univariate analysis indicated that age, type of dialysis, DM, smoking, albumin, i-PTH, ferritin, blood sugar, and Cr were significantly associated with PAD (*p*<0.05 for all). After controlling for age and DM history, multivariate logistic regression analysis indicated that for each 1 mg/dL elevation of Cr, the odds for PAD were 15% lower (OR = 0.85; 95% C.I. = 0.74 to 0.98; *p* = 0.024).

**Table 3:** Univariate and multivariate logistic regression analysis of risk factors associated with PAD in all maintenance dialysis patients (n = 484)

**Variables**	**Univariate**	**Multivariate**	
	**OR (95% C.I.)**	**OR (95% C.I.)**	**P-value**
Age	1.09 (1.07, 1.12)*	1.08 (1.05, 1.11)	<0.001*
Gender			
Male vs. Female	1.42 (0.89, 2.26)		
BMI	0.98 (0.92, 1.05)		
Type of dialysis			
HD vs. PD	5.53 (2.18, 14.04)*	1.83 (0.64, 5.21)	0.258
Duration of Dialysis	0.99 (0.99, 1.01)		
DM			
Yes vs. No	4.46 (2.74, 7.24)*	2.88 (1.58, 5.25)	0.001*
HT			
Yes vs. No	0.95 (0.57, 1.60)		
Hyperlipidemia			
Yes vs. No	1.31 (0.83, 2.09)		
Smoking			
Yes vs. No	1.77 (1.08, 2.91)*	1.37 (0.74, 2.54)	0.322
Daugirdes Kt/V	0.68 (0.36, 1.26)		
Hct	1.02 (0.97, 1.08)		
TCO_2_	1.00 (0.92, 1.08)		
Albumin	0.47 (0.26, 0.85)*	0.98 (0.45, 2.17)	0.963
i-PTH	0.98 (0.97, 0.99)*	0.99 (0.98, 1.01)	0.307
Ferritin	1.00 (1.00 1.01)*	1.00 (0.99, 1.01)	0.706
Cholesterol	1.00 (0.99, 1.01)		
TG	1.00 (0.99, 1.01)		
Blood sugar	1.00 (1.00, 1.01)*	1.00 (0.99, 1.01)	0.383
Cr	0.72 (0.65, 0.80)*	0.85 (0.74, 0.98)	0.024*
Uric Acid	0.87 (0.73, 1.05)		
Ca	1.00 (0.76, 1.33)		
P	0.85 (0.71, 1.01)		
Ca×P	0.99 (0.97, 1.01)		

* Statistically significant (P<0.05).

Next, we used the same univariate and multivariate methods to separately analyze the risk factors for PAD in HD patients (Table 4) and PD patients (Table 5). Among HD patients, advanced age and history of DM were independently associated with PAD. Among PD patients, there were no factors significantly associated with PAD.

**Table 4:** Univariate and multivariate logistic regression analysis of risk factors associated with peripheral artery disease (PAD) in hemodialysis (HD) patients (n = 380)

**Variables**	**Univariate**	**Multivariate**	
	**OR (95% C.I.)**	**OR (95% C.I.)**	**P-value**
Age	1.10 (1.07, 1.13)*	1.09 (1.06, 1.13)	<0.001*
Gender			
Male vs. Female	1.36 (0.84, 2.22)		
BMI	0.99 (0.92, 1.07)		
Duration of Dialysis	0.99 (0.99, 1.01)		
DM			
Yes vs. No	4.05 (2.43, 6.74)*	3.36 (1.80, 6.25)	<0.001*
HT			
Yes vs. No	1.07 (0.61, 1.87)		
Hyperlipidemia			
Yes vs. No	1.42 (0.87, 2.31)		
Smoking			
Yes vs. No	1.46 (0.87, 2.45)		
Daugirdes Kt/V	1.15 (0.58, 2.26)		
Hct	1.01 (0.95, 1.07)		
TCO_2_	1.17 (1.02, 1.30)*	0.98 (0.85, 1.14)	0.832
Albumin	0.33 (0.17, 0.65)*	0.99 (0.43, 2.27)	0.981
i-PTH	0.99 (0.98, 1.00)*	1.00 (0.99, 1.00)	0.213
Ferritin	1.00 (0.99, 1.01)		
Cholesterol	1.00 (0.99, 1.01)		
TG	1.00 (0.99, 1.01)		
Blood sugar	1.00 (1.00, 1.01)*	1.00 (0.99, 1.01)	0.322
Cr	0.73 (0.65, 0.82)*	0.86 (0.74, 1.01)	0.059
Uric Acid	0.81 (0.67, 0.99)*	1.06 (0.83, 1.37)	0.626
Ca	1.14 (0.83, 1.55)		
P	0.92 (0.77, 1.10)		
Ca×P	0.99 (0.98, 1.01)		

* Statistically significant (p<0.05).

**Table 5:** Logistic regression analysis of risk factors associated with peripheral artery disease (PAD) in peritoneal dialysis (PD) patients (n = 104)

**Variables**	**Univariate**
	**OR (95% C.I.)**
Age	1.03 (0.96, 1.10)
Gender	
Male	1.12 (0.18, 6.70)
Female	1.00
BMI	0.90 (0.69, 1.18)
Duration of Dialysis	1.00 (0.98, 1.02)
DM	
No	1.00
Yes	3.22 (0.50, 20.74)
HT	
No	1.00
Yes	0.23 (0.04, 1.43)
Hyperlipidemia	
No	1.00
Yes	1.66 (0.27, 10.37)
Smoking	
No	1.00
Yes	4.41 (0.67, 28.96)
Daugirdes Kt/V	1.44 (0.17, 12.15)
Hct	1.02 (0.82, 1.21)
TCO_2_	1.02 (0.72, 1.45)
Albumin	0.46 (0.06, 3.80)
i-PTH	0.99 (0.98, 1.01)
Ferritin	1.00 (0.99, 1.01)
Cholesterol	1.01 (0.99. 1.03)
TG	1.00 (0.99, 1.01)
Blood sugar	1.00 (0.99, 1.01)
Cr	0.76 (0.54, 1.07)
Uric Acid	0.74 (0.31, 1.76)
Ca	1.54 (0.56, 4.25)
P	0.45 (0.19, 1.05)
Ca×P	0.94 (0.87, 1.02)

## Discussion

We determined the prevalence of PAD in 484 Taiwanese ESRD patients from a single medical center and compared the characteristics of patients who were on HD with those on PD. Our results indicate that advanced age, presence of diabetes mellitus, and low serum creatinine were independently associated with the presence of PAD. Advanced age was the most significant risk factor for PAD (NonPAD: 55.52 ± 13.10, PAD: 68.66 ± 10.54, *p <* 0.001). The inverse association of serum Cr and PAD may seem counter-intuitive. We suggest that this may be due to the lower muscle mass or frailty of these patients. The higher serum Ca, P, and Ca×P were all significantly higher in PD patients, possibly because HD patients were usually on more restrictive diets.

Previous studies of Western populations indicated that PAD is much more common in patients with ESRD (17-48%) than in the general population [14]. The documentation of declining ABI measurements in patients with suspected or known PAD can be an effective way to chart the patient prognosis. Previous studies indicated that this test has a sensitivity greater than 90% and a specificity of 95% for the diagnosis of PAD [15,16]. In particular, one study of 606 patients (mean age: 62years; 68% male) reported that major one-year decline in resting and post-exercise ABI were associated with all-cause mortality, cardiac events, stroke, kidney failure, and PAD [17].

Our study and other studies of PAD examined patients undergoing HD. However, a previous study of Spanish patients with chronic renal failure stages IV and V who were not undergoing dialysis reported that 19% of patients had PAD or other vascular pathologies and that the 5-year mortality rate was higher in patients with PAD than in those without PAD (64% *vs.* 20%) [18]. In addition, a previous study of Japanese subjects with type-2 diabetes mellitus who were not undergoing HD reported that chronic kidney disease had a more significant impact on PAD than metabolic syndrome [19]. Finally, a study of U.S. subjects with no renal abnormalities (participants in the Iron [Fe] and Atherosclerosis Study Study [FeAST], Veterans Affairs [VA] Cooperative Study [CSP] #410) reported significant associations between the levels of serum ferritin, inflammatory biomarkers, and mortality in patients with PAD [20].

Another interesting finding of the present study is that the risk factors for PAD were different in HD patients and PD patients. In particular, pooling all patients together indicated that the risk factors for PAD were similar to those reported in the literature (Table 3). However, separate analysis of the two groups indicated that the risk factors in the HD group remained almost the same as for all patients (Table 4), but that there were no significant risk factors for the PD patients (Table 5). This indicates that further study of additional factors may be needed to better understand the risk for PAD in PD patients.

Our study has several limitations. First, this was a retrospective study, so there may have been errors due to confounding and bias. Second, the study period was only four months, so we cannot make any conclusions about the long-term outcomes. Third, all of our patients were from a single medical center in Taiwan, so our results may not be generalizable to other medical centers in Taiwan or elsewhere. Fourth, our use of ABI for diagnosis of PAD may have led to some misdiagnosis in patients with vessel calcification, leading to false negative diagnoses (type II errors). This limitation could have been overcome by use of toe plethysmography. Finally, we had no records of C-reactive protein, and use of statins and aspirin. Nonetheless, many of the factors which we found increased the risk of PAD can be modified by life style changes. A recent review of PAD noted the importance of risk-factor modification and appropriate pharmacological management for management of PAD [21]. Interestingly, a recent study of PAD in a multi-ethnic Asian population reported that pulse pressure, renal impairment, and a past history of stroke (in addition to traditional risk factors) were important determinants of PAD [22].

## Conclusions

The present retrospective study of Taiwanese ESRD patients from a single center indicated that the overall prevalence of PAD was 18.2%. The prevalence of PAD was greater in the HD group than the PD group, presumably because the PD group was younger and had a lower prevalence of diabetes mellitus. The risk factors associated with PAD were increased age, diabetes mellitus, longer duration of dialysis, low serum creatinine, hyperlipidemia, and elevated serum ferritin. Most of the risk factors for PAD were specific to HD, and no analyzed factor was significantly associated with PAD in PD patients.

## Competing interests

The authors declare that they have no competing interests.

## Authors’ contributions

C-CL and C-FP are guarantors of the integrity of the entire study; C-CL, C-JW, and C-FP are responsible for study concepts, study design, and definition of intellectual content; C-CL and C-FP are responsible for literature research; all authors are responsible for clinical studies and data acquisition; C-CL, C-JW, L-HC, and C-FP are responsible for data analysis and statistical analysis; C-CL and C-FP are responsible for manuscript preparation, manuscript editing, and manuscript review. All authors read and approved the final manuscript.

## Pre-publication history

The pre-publication history for this paper can be accessed here:

http://www.biomedcentral.com/1471-2369/13/100/prepub
